# Serum inflammatory profiles in cystic fibrosis mice with and without *Bordetella pseudohinzii* infection

**DOI:** 10.1038/s41598-021-97033-9

**Published:** 2021-09-02

**Authors:** Paul M. Litman, Alexander Day, Thomas J. Kelley, Rebecca J. Darrah

**Affiliations:** Department of Genetics and Genome Sciences, Case Western Reserve University, Cleveland, OH 44106 USA

**Keywords:** Cystic fibrosis, Cytokines, Infection, Inflammation

## Abstract

Cystic fibrosis (CF) is an autosomal recessive disease caused by dysfunctional cystic fibrosis transmembrane conductance regulator (CFTR) protein, and is marked by an accumulation of mucus in affected airways resulting in persistent infection and chronic inflammation. Quantitative differences in inflammatory markers have been observed in CF patient serum, tracheal cells, and bronchoalveolar lavage fluid, in the absence of detectable infection, implying that absent CFTR function alone may result in dysregulated immune responses. To examine the relationship between absent CFTR and systemic inflammation, 22 analytes were measured in CF mice (F508del/F508del) sera using the MSD multiplex platform. Pro-inflammatory cytokines IL-2, TNF-α, IL-17α, IFN-γ, IL-1β, and MIP-3α are significantly elevated in infection-naïve CF mice (*p* < 0.050). Anti-inflammatory cytokines IL-10 and IL-4 are also significantly increased (*p* = 0.00003, *p* = 0.004). Additionally, six general markers of inflammation are significantly different from non-CF controls (*p* < 0.050). To elucidate the effects of chronic infection on the CF inflammatory profile, we examined CF mice exposed to spontaneous *Bordetella pseudohinzii* infections. There are no statistical differences in nearly all inflammatory markers when compared to their infection-naïve CF counterparts, except in the Th2-derived IL-4 and IL-5 which demonstrate significant decreases following exposure (*p* = 0.046,* p* = 0.045). Lastly, following acute infection, CF mice demonstrate elevations in nearly all inflammatory markers, but exhibit a shortened return to uninfected levels over time, and suppression of Th1-derived IL-2 and IL-5 (*p* = 0.043, *p* = 0.011). These results imply that CF mice have a persistent inflammatory profile often indistinguishable from chronic infection, and a dysregulated humoral response during and following active infection.

## Introduction

Cystic fibrosis (CF) is a complex genetic disease that affects approximately 1 in every 3400 births in the United States, and is the most common lethal genetic disease among Caucasian populations^1^. CF is caused by mutations in the cystic fibrosis transmembrane conductance regulator (CFTR) gene that result in dysfunctional CFTR protein^2,3^, the most common mutation being F508del. CF affects almost all organ systems but most severely affects the lungs, gut, and endocrine system. In the lungs, CF is marked by an accumulation of mucus in the epithelial cell layer^4,5^, resulting in spontaneous and progressive infections and severe inflammation in the lungs. The cycle of infection and inflammation results in decreased lung function, which is the leading cause of morbidity and mortality in individuals with CF^6,7^.

Inflammation is present early in CF, even prior to identified infection, and persists throughout the course of the disease^8^. In attempts to identify the underlying causes for increased inflammation, researchers have measured proinflammatory and anti-inflammatory cytokines in the epithelial lung fluid (ELF), bronchoalveolar lavage fluid (BALF), sputum, and serum of CF patients of varying ages and various states of disease progression^9,10^. CF patients infected with *Pseudomonas aeruginosa* or *Burkholderia cepacia* show elevated levels of proinflammatory cytokines IL-1β, IL-8, IL-6 and TNF-α, as well as depleted levels of the anti-inflammatory cytokine IL-10 in their BALF and ELF compared to healthy controls^9^. Interestingly, elevated proinflammatory markers were also found in CF infant BALF as well as in naïve tracheal CF airway grafts, both in the absence of any detectable infection^11^. These findings imply that CF-specific inflammation may not be due solely to chronic infection, but can be a direct consequence of absent or dysfunctional CFTR protein. A similar increase in proinflammatory markers was also found in the serum of infected CF patients when compared to non-CF controls^12,13^. Proteomic studies also reveal the presence of increased inflammatory markers in serum samples of CF patients^14–16^. Human studies on serum cytokines suggest chronic systemic inflammation, but it is difficult to determine if this inflammation is due to years of chronic infection or innate changes due to the loss of CFTR function. Mouse models of CF offer an opportunity to determine the role of CFTR function in controlling measures of systemic inflammation.

The F508del CF mouse has been a valuable tool for studying the effects of CF-dependent lung disease. Previous research has demonstrated that the F508del mouse exhibits a significant decrease in lung function compared to C57BL6, WT mice in the absence of infection^17^, such as decreased lung compliance and increased elasticity, resulting in more effort to move air compared to non-CF mice^18,19^. To assess the inflammatory response associated with non-functional CFTR in mice, data on a small subset of pro- and anti-inflammatory cytokines have been reported in CF mice^20^, though most exclusively utilize BAL fluid. Relatively little work has been done to determine whether circulating serum levels of these cytokines correlate with the changes in BALF reported in the CF mouse lung.

Cystic fibrosis disease progression includes an excessive proinflammatory response in the presence of infection. While CF mice are not known to exhibit spontaneous *Pseudomonas aeruginosa* infections, it has been demonstrated that CF mice are prone to developing spontaneous *Bordetella pseudohinzii* lung infections^21^. These infections appear to be directly related to the absence of CFTR, as CF mice are far more susceptible to the pathogen and less able to resolve their infection than their non-CF counterparts^21^. These infections result in a significant decline in breathing rate, but little work has been done to examine the inflammatory response to these spontaneous infections.

To determine the role of loss of CFTR on serum-specific markers of inflammation, we measured twenty-nine cytokines and chemokines involved in multiple aspects of inflammation in F508del CF and non-CF mice. We measured changes in cytokine levels as a response to spontaneous lung infection in mice exposed to *B. pseudohinzii*. Lastly, we tracked the inflammatory response during an induced infection of *B. pseudohinzii* over the course of 2 weeks to determine the inflammatory response in the presence of acute infection in the CF mouse.

## Materials and methods

### CF and non-CF mouse models

A well-described congenic mouse model for cystic fibrosis, homozygous for the F508del *Cftr* mutation (*Cftr*^*tm1kth*^), was utilized for this study^17,18^. These mice are congenic on the C57BL/6J background and are backcrossed every 5 generations to minimize genetic drift. As a result, wild-type C57BL/6J mice were used as non-CF controls. Adult mice (at least 8 weeks of age), half male and half female, were used in the study and were allowed unrestricted access to chow (Harlan Teklad 7960; Harlan Teklad Global Diets, Madison, WI) and sterile water with the osmotic laxative, Colyte (Schwarz Pharma, Milwaukee, WI). The mice were maintained on a 12 h light, 12 h dark cycle at a mean ambient temperature of 22 °C throughout the course of the study.

### Statement

All methods utilized were performed in accordance with the relevant guidelines and regulations. Specifically, The Institutional Animal Care and Use Committee of Case Western Reserve University approved the experimental protocols used. All mouse experiments were reported in accordance to the ARRIVE guidelines^22^, and are consistent with the American Veterinary Medical Association (AVMA) Guidelines for the Euthanasia of Animals (2020).

### Blood extraction and serum isolation

Whole blood was drawn postmortem in serum separating microtainer tubes (BD) and centrifuged at 10,000 rpm for 5 min. After centrifugation, serum was pipetted into sterile microcentrifuge tubes and stored immediately at − 80 °C.

### *B. pseudohinzii* ELISA

Mouse serum was diluted 1:50, applied in duplicate to pre-coated mouse *B. hinzii* antigen wells (XpressBio, Cat. No. 595-470C), and incubated at 37 °C for 30 min according to manufacturer’s instructions. Wells were washed and incubated with peroxidase conjugate at 37 °C for 45 min, incubated with ABTS substrate for 30 min, and read at 405 nm on an absorbance plate reader. Results were considered positive if the average absorbance in duplicate minus the negative antigen controls > 0.100. Note: Manufacturer’s protocol states wells are to be considered positive with an absorbance > 0.300, however evidence from our lab has demonstrated that mice positive for *B. pseudohinzii* (verified via plethysmography and BAL cultures) should be considered positive by ELISA at this lower cutoff, and still remain distinguishable from negative ELISA results.

### Meso scale discovery (MSD) MULTI-SPOT mouse inflammatory panel assay system

Manufacturer-supplied cytokine standards and twofold dilutions of mouse serum were applied in duplicate to pre-coated V-plex wells (MSD Mouse Cytokine 29-plex, MSD Mouse Proinflammatory Panel 1, K15267D-1, K15048D-1) and incubated with shaking at room temperature for 2 h. Each plate was then washed, blotted dry, and incubated with detection antibody for 2 h. Following incubation, read buffer was added and each plate was immediately read using a Meso Scale Discovery QuickPlex SQ 120 and data were analyzed with the supplied software. The 29 separate assays were multiplexed across three plates in total.

### *Bordetella pseudohinzii* infection

Infection was examined in two separate populations of mice. The first were CF mice developing natural, spontaneous infections at some point during their lifetime. These were mice living naturally in their colonies that tested positive for *B. pseudohinzii* antibodies following sacrifice. ELISA-based approaches are not indicative of active infection, and we can only confirm that these mice were at one point exposed to the pathogen and mounted an immune response.

The second population of mice were verified infection-naïve before being intentionally infected with an active strain of *B. pseudohinzii*. Optical density readings were used to plot a growth curve of the *B. pseudohinzii* culture to determine the log-phase of the pathogen. The inoculum was taken from the culture once log-phase was reached to ensure pathogen viability. Isoflurane-anesthetized mice were inoculated with a 5 × 10^6^ bolus of *B. pseudohinzii* intranasally. Whole blood was extracted from the mice 48 h and 2 weeks after infection and immediately placed in serum separator microtainers for further analyses.

### Statistics

Hypothesis testing was performed using single factor ANOVA (significant at *p* < 0.05). As this study was exploratory in nature, and the number of comparisons required with a multiplexed strategy are numerous, we did not adjust our initial p-value. We instead followed individual ANOVAs with the conservative post-hoc Tukey’s Honest Significant Difference to determine significance within each group and reduce error within the individual tests. While this can inflate type 1 error caused by multiple comparisons, it gives a clear direction of significance among the multiple groups. Extreme outliers that fell outside of the upper or lower limit of the data set (1.5 times the interquartile range outside of the first or third quartiles) were removed and not included in the ANOVA. Male and female results were analyzed individually, and as there was no significant difference in the direction or magnitude of change, the data were combined for additional statistical power.

## Results

### Serum-specific markers of inflammation in CF mice

To examine the serum-specific inflammatory profile in F508del mice, twenty-nine different cytokines were measured in a high-throughput, multiplexed colorimetric system. Of the twenty-nine cytokines measured, data were recorded from 22, with the remaining 7 falling below a detectable range when compared to the standard curve. Only cytokines in which a detectible concentration was obtained are presented. The measured cytokines are divided into three categories: proinflammatory, anti-inflammatory, and general inflammatory markers.

### Non-CF and CF proinflammatory markers

**Figure 1:**
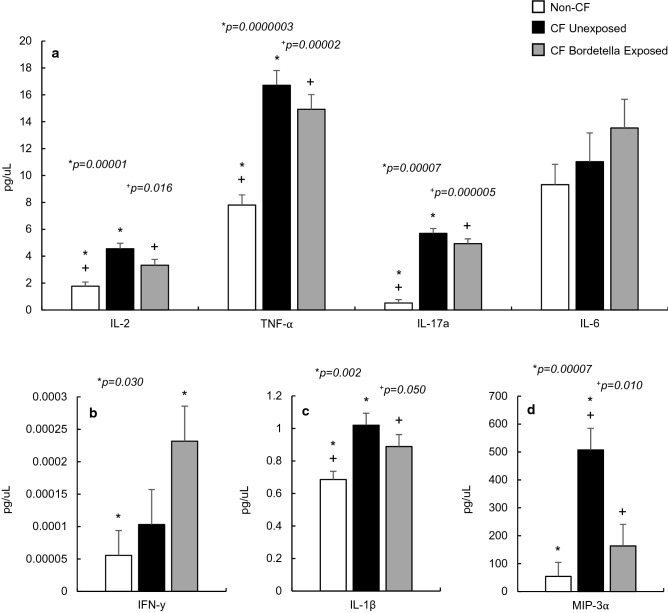
Proinflammatory cytokine concentrations in non-CF mouse serum (n = 20), *Bordetella pseudohinzii* unexposed CF mice (n = 10), and *B. pseudohinzii* exposed CF mice (n = 10). All analytes are significantly elevated in CF unexposed sera when compared to non-CF controls, with the exception of IL-6 (**a**) and IFN-γ (**b**). Serum concentrations remain elevated in *B. pseudohinzii* exposed mice compared to non-CF mice, but were not significantly different from their unexposed CF counterparts with the exception of MIP-3α (**d**) which was reduced in exposed mice. IFN-γ, IL-1β, and MIP-3α (**b**–**d**) are displayed separately to visualize differences in scale.

Serum proinflammatory markers for IL-1β, IL-2, TNF-α, IL-17α, IL-6, IFN-γ, and MIP-3α were recorded in twenty CF and twenty age-matched non-CF mice (Fig. 1). Of these seven analytes, nearly all were significantly elevated (*p* < 0.050, Fig. 1a–d) in CF mice compared to the non-CF with the exception of IFN-γ and IL-6. Previous work in CFTR-deficient mice have shown similar increases in BALF proinflammatory markers, especially in Th1 cell-secreted cytokines directly related to cell mediated immunity, macrophage presence, and activity^23^, which we see recapitulated in serum for IL-2 and TNF-α (Fig. 1a) in the F508del mice. Of note, previously reported increases of IL-6 in BALF were not recapitulated in the serum (Fig. 1a). We also observed a significant increase of MIP-3α, being increased nearly sevenfold (*p* = 0.00007, Fig. 1d) in the CF mice.

### Non-CF and CF anti-inflammatory markers

**Figure 2:**
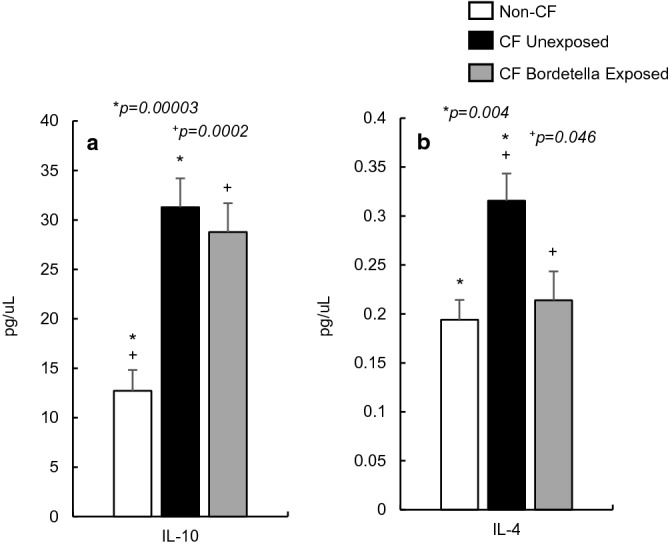
Anti-inflammatory cytokine concentrations in non-CF (n = 20), CF unexposed (n = 10), and CF *Bordetella pseudohinzii* exposed (n = 10) mouse serum. Baseline levels of IL-10 and IL-4 are significantly elevated in CF unexposed sera versus Non-CF controls. Following *B. pseudohinzii* exposure, IL-10 (**a**) remains elevated compared to non-CF controls (+), but is not significantly different from the unexposed CF mice. IL-4 (**b**) is significantly reduced in CF exposed mice to near-non-CF levels (+).

Two predominant anti-inflammatory cytokines, Th1-derived IL-10 and Th2-derived IL-4, were measured in CF and non-CF sera (Fig. 2). Previous studies in the CF mouse lung report decreases of anti-inflammatory markers^10,24^. However, in CF sera, we showed a marked *increase* in IL-10 levels (*p* < 0.05) (Fig. 2a), independent of infection.

We observed similar results when examining IL-4, which serves as a positive feedback cytokine, downregulating IFN-γ and TNF-α, and plays a vital role in antigen response. In CF serum, corresponding to the increase in its effector proinflammatory cytokines, there is a significant increase in IL-4 serum concentration when compared to non-CF controls (*p* < 0.05) (Fig. 2b).

### Non-CF and CF general inflammation markers

**Figure 3:**
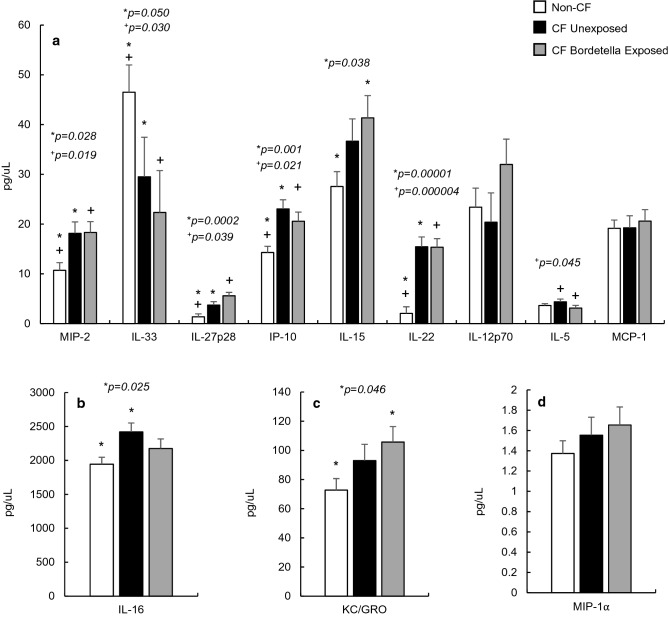
General inflammatory marker concentrations in non-CF (n = 20), CF unexposed (n = 10), and CF *Bordetella pseudohinzii* exposed (n = 10) mouse serum. MIP-2, IL-27p28, IP-10, IL-22 (**a**), and IL-16 (**b**) were significantly elevated in CF mice. Notably, IL-33 (**a**) is the only inflammatory marker that showed a significant baseline reduction in CF mice when compared to non-CF controls. IL-5 (**a**) displays a significant reduction (+) in concentration following *B. pseudohinzii* exposure when compared to unexposed CF counterparts. There are no other differences in inflammatory markers between unexposed and exposed CF mice, though all remain elevated as compared to non-CF controls. IL-16, KC/GRO, and MIP-1α (**b**–**d**) are displayed separately to visualize differences in scale.

The last group of analytes are neither proinflammatory nor anti-inflammatory markers, but are often pleiotropic in nature, and are regulated more specifically based on the inflammatory event. These markers include the C-X-C chemokines MIP-1α, MIP-2, IP-10, and keratinocyte chemoattractant/growth regulated oncogene KC/GRO, the monocyte chemoattractant protein MCP-1, and members of multiple Interleukin families in IL-5, IL-12p70, IL-15, IL-16, IL-22, IL-27p28, and IL-33 (Fig. 3a–d). There was a significant difference between CF and non-CF serum concentrations for 6 of the 12 general inflammatory markers, with MIP-2, IL-27p28, IP-10, IL-22, and IL-16 demonstrating a significant increase when compared to the non-CF controls (*p* < 0.050, Fig. 3a,b). Notably, IL-33 (Fig. 3a) is the only inflammatory marker tested that demonstrated a significant *decrease* (*p* = 0.050) in serum levels in CF mice compared to non-CF.

### Effects of spontaneous infection on markers of inflammation

CF mice in our facility have been shown to develop spontaneous *B. pseudohinzii* infections, with roughly 30% of CF mice developing the infection over the course of their lives compared to only 2% of non-CF mice in a mixed population^21^. In addition, CF mice have difficulty clearing the infection, requiring 8–12 weeks or longer to resolve an infection as opposed to non-CF mice, requiring only 1–3 weeks as confirmed by plethysmography and BAL fluid culture^21^. To determine the effect of spontaneous lung infection on serum inflammatory markers, we compared a subset of our CF population that had been exposed to the pathogen, testing positive for *B. pseudohinzii* antibodies, against our infection-naïve mice as determined by ELISA (Table 1).

**Table 1:** *B. pseudohinzii* ELISA. The CF mouse population was differentiated into exposed and unexposed groups based the presence of antibodies towards the *B. pseudohinzii* antigen. Non-CF and CF mice were also verified via ELISA to be infection-naïve before intentional infection with *B. pseudohinzii*. Results were considered positive at a > 0.100 absorbance threshold.

Group	n	Avg. ELISA absorbance	Range
Non-CF	20	0.013	− 0.009 to 0.059
CF unexposed	10	0.031	0.001 to 0.064
CF exposed	10	0.326	0.112 to 0.700
Non-CF pre-infection	12	0.022	0.008 to 0.039
CF pre-infection	12	0.028	0.015 to 0.049

### *B. pseudohinzii* exposure and proinflammatory markers

CF mice exposed to *B. pseudohinzii* demonstrate no significant difference from their unexposed CF-counterparts in nearly all proinflammatory markers, and are significantly elevated compared to non-CF mice (Fig. 1a–d). A notable exception was MIP-3α (Fig. 1d), which displayed a reduction in serum concentration following exposure when compared to the unexposed CF mice (*p* = 0.010), and not statistically dissimilar to the non-CF mice. Only IFN-γ serum concentrations were significantly elevated as a result of *B. pseudohinzii* exposure beyond that of their CF counterparts (*p* = 0.030).

While exposure does not indicate the presence of an active infection, this lack of difference is of particular interest given the persistence of lung pathophysiology, the length of infection, and difficulties in pathogen clearance CF mice exhibit during *B. pseudohinzii* infection. Based on this, it is reasonable to assume a number our exposed mice were still immunologically challenged, but we see no increased proinflammatory response beyond what is seen in a CFTR-deficient model alone.

### *B. pseudohinzii* exposure and anti-inflammatory markers

Contrary to previous findings in the CF mouse lung during *P. aeruginosa* infection^24^, IL-10 remains significantly increased in serum of the CF mice exposed to *B. pseudohinzii*, but there is no marked difference from the infection-naive CF control mice (Fig. 2a). Surprisingly, IL-4 is significantly reduced (*p* = 0.004) in exposed CF mice compared to their unexposed CF counterparts, suggesting that there is a possible reduction in the Th2 humoral response, mediated by IL-4, in the presence of *B. pseudohinzii* infection.

### *B. pseudohinzii* exposure and general inflammation markers

There are no significant differences in serum levels of general inflammatory markers between CF unexposed and *B. pseudohinzii* exposed mice, except in the Th2-derived IL-5 (Fig. 3a, *p* = 0.045). This finding is particularly remarkable, as it, along with IL-4, represents another marker of humoral immunity being downregulated following infection.

In multiple cases [IL-15, IL-12p70, and MCP-1 (Fig. 3a), KC/GRO (Fig. 3c), MIP-1α (Fig. 3d)], there are non-significant elevations in the exposed CF mice compared to non-exposed. Only in the case of IL-15 and KC/GRO do these elevations, caused by *B. pseudohinzii* exposure, lead to a significant increase in serum concentration (*p* = 0.038, 0.046) (Fig. 3a,c).

### Effects of active infection on markers of inflammation

Despite the long infection window in CF mice, pathogen exposure as determined by antibody-based ELISA approaches doesn’t necessarily indicate an active infection, which could explain the notable lack of increased proinflammatory cytokines in CF mice exposed to *B. pseudohinzii,* when compared to infection-naive, CF controls. To determine whether active infection will alter the inflammatory profile, we inoculated non-CF and CF mice intranasally with *B. pseudohinzii* cultures and measured a subset of our inflammatory markers at 48-h and 2-weeks post intentional infection as compared to infection-naïve controls (Table 1).

### Active *B. pseudohinzii* infection and proinflammatory markers

Although non-CF mice rarely contract spontaneous *B. pseudohinzii* infections, they were included as a control to visualize differences in inflammatory responses following intentional infection. In the non-CF mice, there is a visual elevation of all analytes at 48 h, though only two of five measured cytokines, IL-6 and TNF-α, were significantly elevated (*p* = 0.002, *p* = 0.049 respectively), and remained elevated 2 weeks post infection (*p* = 0.010, *p* = 0.044).

**Figure 4:**
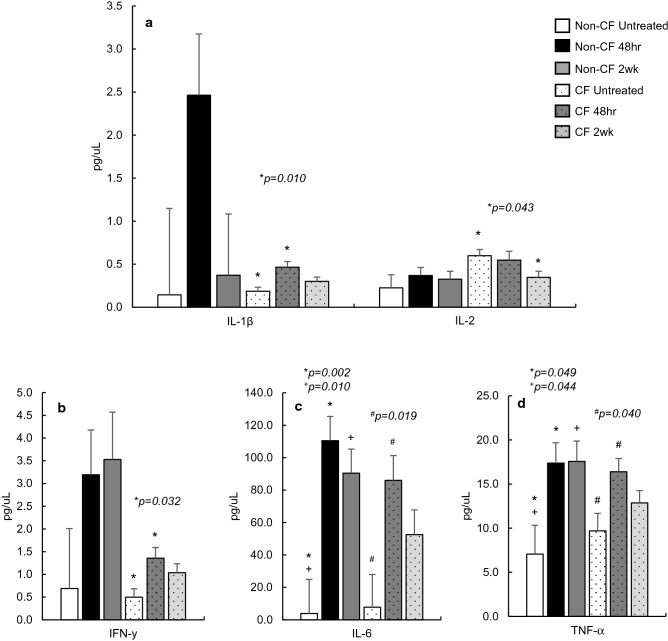
Proinflammatory marker concentrations in untreated Non-CF (n = 4) and CF (n = 4) mice, and then following *Bordetella pseudohinzii* inoculation at 48 h and 2 weeks post-infection (Non-CF n = 8, CF n = 8). In Non-CF mice, there is a significant elevation in IL-6 and TNF-α, both of which remain elevated at 2 weeks post-infection. In CF mice, all but IL-2 exhibit a significant increase in serum concentration at 48 h post-infection as compared to uninfected CF controls, and drop after 2 weeks to near uninfected levels (**a**–**d**). In CF mice, IL-2 displays as light reduction post-inoculation and sharply declines 2 weeks post-infection (**a**). IFN-γ, IL-6, and TNF-α (**b**–**d**) are displayed separately to visualize differences in scale.

In the CF mice, within 48-h *of B. pseudohinzii* inoculation, there is a significant spike in IL-1β (*p* = 0.010), IFN-γ (*p* = 0.032), IL-6 (*p* = 0.019), and TNF-α (*p* = 0.040) serum levels (Fig. 4a–d), well beyond the infection naïve controls. Though, when measured again 2-weeks post-infection, there is a notable decrease in serum levels of all markers, falling back to concentrations not significantly different than their uninfected CF counterparts. Interestingly, IL-2 was dramatically reduced, resulting in serum concentrations significantly lower than the untreated CF controls (*p* = 0.043) at 2 weeks post-infection.

### Active *B. pseudohinzii* infection and anti-inflammatory markers

**Figure 5:**
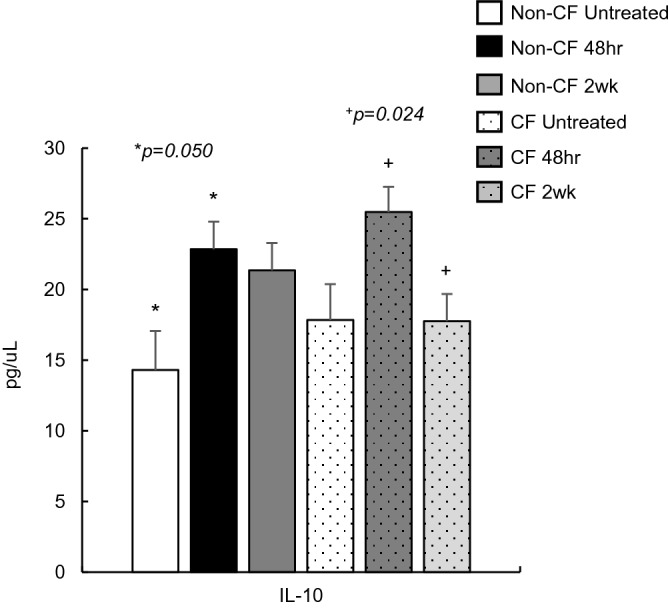
Anti-inflammatory marker concentrations in untreated Non-CF (n = 4) and CF (n = 4) mice, and then following *Bordetella pseudohinzii* inoculation at 48 h and 2 weeks post-infection (Non-CF n = 8, CF n = 8). In both Non-CF and CF, there is a significant increase in serum IL-10 concentrations at 48 h post inoculation. IL-10 remains elevated in the Non-CF mice at 2 weeks post-infection, while in CF mice, drops significantly after 2 weeks to near untreated levels.

Of the two anti-inflammatory markers (IL-10 and IL-4), only IL-10 was within a detectable range following *B. pseudohinzii* inoculation (Fig. 5). In both non-CF and CF mice, there is a significant increase in IL-10 serum concentrations at 48 h post-infection (*p* = 0.050,* p* = 0.024). After 2 weeks, levels remain elevated in the non-CF mice, but are dramatically reduced in the CF mice, significantly lower than at the 48-h time point (*p* = 0.024), and similar to their untreated counterparts.

### Active *B. pseudohinzii* infection and general inflammation

**Figure 6:**
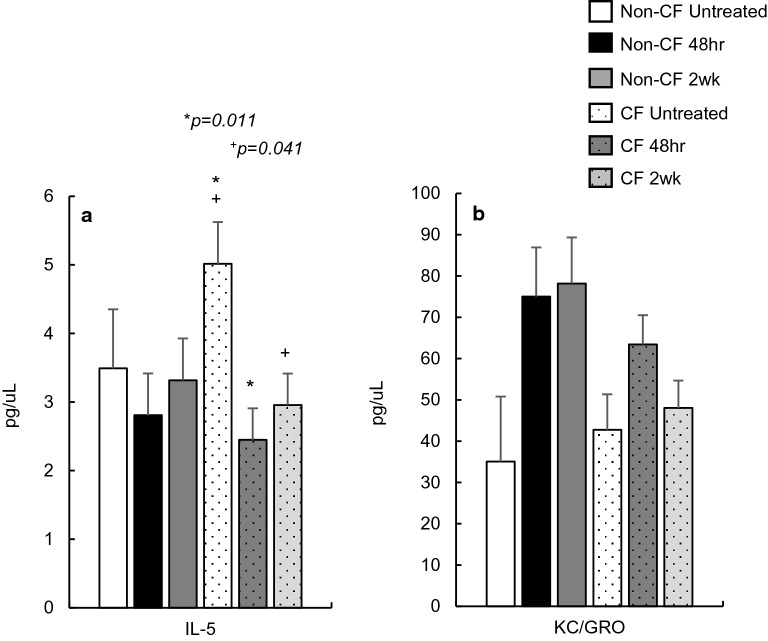
General inflammatory marker concentrations in untreated Non-CF (n = 4) and CF (n = 4) mice, and then following *Bordetella pseudohinzii* inoculation at 48 h and 2 weeks post-infection (Non-CF n = 8, CF n = 8). In CF, IL-5 shows a significant decrease in concentration 48 h post inoculation (**a**), and remains significantly lower 2 weeks post-infection. KC/GRO shows a non-significant elevation 48 h post inoculation before falling to untreated levels after 2 weeks (**b**).

Of the previous general inflammatory markers, we looked specifically at IL-5 and KC/GRO concentration during active infection. Serum levels of IL-5 are reduced in non-CF mice, and, consistent with our exposure data, are significantly reduced in CF mice (*p* = 0.011) at 48 h post inoculation with *B. pseudohinzii* (Fig. 6a). Concentrations remain below untreated CF mice for the 2-week duration of infection (*p* = 0.041). Also consistent with exposure data, KC/GRO shows an elevation in serum concentration at 48-h post inoculation, then falls to near CF untreated levels after 2 weeks (Fig. 6b), though non-significantly.

## Discussion

In humans with CF, the lung environment is highly complex, characterized by fibrosis, excessive mucus, airway dehydration, neutrophil accumulation, monocyte and macrophage inefficiency^25^, and persistent bacterial infection, which leads to a decline in pulmonary function. This environment results in a state of chronic inflammation, and proinflammatory markers in the lungs such as TNF-α, IL-1β, IL-6, and IL-8 are consistently reported to be elevated in BAL fluid, while anti-inflammatory cytokines are often reduced^9,10^. When this balance is dysregulated, the result can either be a deleterious, excessive proinflammatory response, or a potential immunosuppression and increased susceptibility to secondary infection by opportunistic pathogens^26^.

In this study, we first sought to determine what baseline differences existed in a wide variety of pro-inflammatory, anti-inflammatory, and general inflammatory cytokines in the F508del CF mouse in the absence of detectable infection to determine the role of CFTR function in the regulation of systemic inflammation. These markers represent cytokines and chemokines across multiple families that regulate cell mediated inflammatory responses (T-helper type 1-derived) or adaptive immunity (T-helper type 2-derived) in response to pathogens. In the F508del CF mouse serum, we have demonstrated an increase in proinflammatory cytokines IL-1β, IL-2, and TNF-α, consistent with previously reported findings in the CF mouse^23,27^. Further, in the CF mouse we have demonstrated significant elevations in serum levels of novel proinflammatory cytokines IL-17α and MIP-3α, and novel pleiotropic, general markers of inflammation MIP-2, IL-27p28, IP-10, IL-16, and IL-22. In Th1-derived, cell mediated immunity, monocytes and macrophages have been previously identified as a key component of the dysregulated immune response in CF, and a loss of CFTR in macrophages contributes to the hyperinflammation seen in CF BALF^28^. Our findings in serum support and expand on this, as nearly every cytokine and chemokine involved with a cell-mediated response (MIP-2, MIP-3α, MCP-1, IL-1β, IP-10, TNF-α) was significantly elevated in the CF mouse, even without detectable infection. Interestingly, there was also a significant increase in Th2-mediated inflammatory cytokines (IL-2, IL-16, IL-22), responsible for B-cell activation and antibody production and isoform conversions. Both Th1- and Th2-derived cytokines can act as effectors in feedback loops within and between themselves to regulate appropriate responses to pathogens. Even in the absence of infection, loss of CFTR alone contributes to significant dysregulation of nearly every cytokine involved in cell mediated and adaptive immunity.

Contrary to previous findings in the CF mouse lung, our serum concentrations of both anti-inflammatory markers IL-10 and IL-4 were also significantly elevated (Fig. 2) in the CF mouse. IL-10, a key immunoregulator during pathogenic challenge, plays a major role in the inhibition of Th1-specific, macrophage-derived proinflammatory cytokines. It is not constitutively expressed, and is found in low levels in serum in the absence of pathogenic stimulation^29^. The excess production of IL-10 in our infection-naïve model might then be partially explained as a response to the overexpression of its proinflammatory inhibition targets. Yet, despite its excess, nearly all proinflammatory markers, with the exception of MIP-3a (Fig. 1d) remain significantly increased in the CF mouse. IL-4 is a pleiotropic anti-inflammatory cytokine that plays a major role regulating humoral immunity, aiding in the polarization of naïve CD4+ Th cells into Th2 effector cells, and via signal transduction, suppresses Th1 cell development^30^. In mice, a lack of IL-4 can result in attenuated airway inflammation, whereas an overexpression can result in the inability to fight infection^31^. We would expect the overabundance of IL-4 present in the CF mice to lead to a suppression of a Th1 cell mediated response, which was not seen. This indicates a possible lack of effect of IL-4 as a traditional anti-inflammatory cytokine in the absence of CFTR. Interestingly, IL-4 has been shown to have a specific pathology in the human lung, directly inducing mucin gene expression and increasing in asthmatic patients^32^. This CF-specific overexpression, then, could be of particular interest, exacerbating an already volatile lung environment through overexpression and inefficiency at its role as an anti-inflammatory in the absence of CFTR.

With a baseline inflammatory panel established, we sought to determine what effects spontaneous lung infection and pathogen exposure would have on the immune response in CF mice. Surprisingly, there is very little change in cell-mediated inflammatory responses following exposure to *B. pseudohinzii* when compared to infection-naïve CF mice, further suggesting that the differences observed are unrelated to chronic infection. Every proinflammatory cytokine involved in monocyte/macrophage regulation and recruitment remain significantly overexpressed in the serum with persistent pathogenic exposure, but are not significantly different from the uninfected CF controls. This indicates that the consistently exaggerated cell-mediated inflammatory state of CF mice cannot be attributed solely to chronic pathogenic exposure, and at some level of exposure, can be indistinguishable from the inflammatory response due to the loss of CFTR alone.

What was striking was the effect of *B. pseudohinzii* exposure to cytokines involved in regulating the Th2-derived, humoral response to infection. CF mice exposed to *B. pseudohinzii* displayed a significant decline in IL-4, and a non-significant reduction in IL-2 (Figs. 1a, 2b) serum levels, both of which serve as the predominate regulators of T-cell proliferation and B-cell activation during an adaptive immune response to infection. Furthermore, after exposure, we see a significant reduction in other notable Th2 mediated cytokines IL-5 and IL-33, and non-significant reductions in IL-16 and IL-17a. Taken together, these data suggest that the loss of CFTR, in conditions of chronic exposure to a pathogen, could potentially interfere with the ability to mount and maintain an adaptive immune response to infection, and in turn could explain the exaggerated, chronic cell-mediated response in the form of overstimulated Th1 effector cells.

Finally, based on the above evidence that CF mice didn’t appear to mount an appropriate, serum-specific proinflammatory response to pathogen exposure, we sought to determine if we could elicit a noticeable immune response following an acute, active infection. Immediately after a *B. pseudohinzii* challenge, both non-CF and CF mice produced a spike in serum-specific proinflammatory markers, significantly higher in the CF mice, and well beyond those of the infection-naïve controls. After 2 weeks, cytokine levels in the non-CF mice appeared to remain elevated as they continued to maintain an active immune response. In contrast, the CF mice immune response declined in nearly all cases at 2 weeks post infection to levels not statistically different than the uninfected CF controls. This timeframe of immune response is consistent with our previous findings demonstrating the CF mouse’s difficulties in clearing an active infection. And, while it can take upwards of 12 weeks for a CF mouse to clear an active *B. pseudohinzii* infection, cytokine concentrations involved in a cell-mediated response fall by nearly half in only the first 2 weeks. During the remaining recovery period, representing a period of chronic infection, the cell mediated inflammatory response is nearly indistinguishable from the inflammation response brought on by a loss of CFTR alone.

Most striking again is the active suppression of the adaptive immune response following active infection, similar to that seen in *B. pseudohinzii* exposed CF mice. Immediately after inoculation, IL-2 levels are lower than our CF controls, and 2 weeks post infection, continue to drop significantly below infection-naïve CF mice (Fig. 4a). Unfortunately, we were unable to report data on IL-4, as it fell below a detectable range in our mouse populations. But, IL-5, which is traditionally produced in response to IL-4 stimulation, is significantly reduced 48-h post infection (Fig. 6a), and remains low throughout the course of infection. We can infer then, as seen in conditions of chronic exposure, there is again a suppression of the adaptive immune response to infection that is produced early during a pathogenic challenge and remains throughout the course of infection. These findings provide some insight into the difficulties CF mice have in infection clearance.

The results described here in CF mice are similar to those observed in individuals with CF. Cytokine analyses from the serum of CF patients indicates an elevation of all cytokines shown to be elevated in this present study^14^, though controlling for infection status was not possible with human studies. Further, studies in humans are complicated by the fact that infection-naïve controls are not realistic. Efforts are ongoing to determine a molecular signature of CF disease status in humans, and the results presented here suggest a similar profile may be obtainable and comparable in CF mouse models^14^, providing a valuable tool for studying CF inflammatory responses in the presence or absence of pathogenic challenge.

Here we have described a serum-specific inflammatory profile in F508del, CF mice. This profile verifies, in sera, previous data exclusive to the lungs, and introduces novel markers of inflammation previously unstudied in the CF mouse. We’ve found significant differences between non-CF and CF mice in the absence of detectable infection, implying these differences are primarily attributable to the loss of CFTR. These differences result in a chronic, exaggerated proinflammatory state, particularly in those cytokines involved in Th1-specific, cell-mediated immunity. Lastly, we have explored the inflammatory response of CF mice during spontaneous infections caused by chronic, *B. pseudohinzii* exposure, and during an active bacterial challenge. We have discovered a downregulation of those cytokines primarily responsible for a Th2-mediated, adaptive immune response during bacterial challenge, and persisting for the duration of recovery. Specific serum cytokine profiles at different stages of infection (undetectable, acute, and chronic) could prove to be a valuable biomarker for assessing patient’s overall health or risk of acute exacerbation. Since cytokine profiles are also dependent on CFTR function, serum cytokines may be an easily accessible biomarker to determine responses to CFTR modulator therapies.

## Data Availability

The datasets generated during and/or analyzed during the current study are available from the corresponding author on reasonable request.
